# Cost effectiveness of clinical associates: A case study for the Mpumalanga province in South Africa

**DOI:** 10.4102/phcfm.v8i1.1218

**Published:** 2016-11-15

**Authors:** Joris Hamm, Petra van Bodegraven, Martin Bac, Jakobus M. Louw

**Affiliations:** 1 Family Medicine Department, University of Pretoria, South Africa

## Abstract

**Background:**

The National Department of Health of South Africa decided to start a programme to train mid-level healthcare workers, called clinical associates, as one of the measures to increase healthcare workers at district level in rural areas. Unfortunately, very little is known about the cost effectiveness of clinical associates.

**Aims:**

To determine, on a provincial level, the cost effectiveness of training and employing clinical associates *and* medical practitioners compared to the standard strategy of training and employing *only* more medical practitioners.

**Methods:**

A literature study was performed to answer several sub questions regarding the costs and effectiveness of clinical associates. The results were used to present a case study.

**Results:**

The total cost for a province to pay for the full training of a clinical associate is R 300 850. The average employment cost per year is R196 329 and for medical practitioners these costs are R 730 985 and R 559 397, respectively.

**Effectiveness:**

Clinical associates are likely to free up the time of a medical practitioner by 50–76%. They can provide the same quality of care as higher level workers, provided that they receive adequate training, support and supervision. Furthermore, they seem more willing to work in rural areas compared to medical practitioners.

**Conclusions:**

The case study showed that training and employing clinical associates is potentially a cost-effective strategy for a province to meet the increasing demand for rural healthcare workers. This strategy will only succeed when clinical associates receive adequate training, support and supervision and if the province keeps investing in them.

## Introduction

South Africa faces challenges with regard to the number and distribution of healthcare workers, especially in rural areas. In the public sector, there is a huge shortage of healthcare workers and the workload for doctors is too high.^1,2^

The National Department of Health (NDoH) decided to start a programme to train mid-level healthcare workers, called clinical associates (ClinAs), as one of the measures to increase healthcare workers at district level in rural areas.^1,3^ Unfortunately very little research is done in the area of mid-level workers, especially in primary health care and developing countries.^4,5,6,7,8^

One of the priorities for further research is the cost effectiveness of mid-level workers in relationship to health gains and compared to other health care workers.^4,5,6,9^

### Aim and objectives

This study will try to determine the cost effectiveness of training and employing ClinAs *and* medical practitioners (MPs) compared to the standard strategy of training and employing *only* more MPs.

This comparison can help a province to decide if and how much, beside training and employing MPs, the province should be investing in training and employing ClinAs, as an additional strategy to reduce the healthcare workers shortage in rural areas.

### Main research question

What is the cost effectiveness for a province in South Africa of investing in training and employing ClinAs, compared to investing in training and employing more MPs?

### Sub questions

We will answer our main questions with the help of the following sub questions:

### Costs

What are the costs for a province to train a clinical associate compared to training a medical practitioner?

What are the costs for a province to employ a clinical associate in comparison to employing a medical practitioner?

### Effectiveness

What is the scope of practice of ClinAs and how does this compare to MPs?

How much medical practitioner time can a clinical associate save?

What is the quality of care provided by ClinAs?

How is the retention of ClinAs in rural areas and how does this compare to MPs?

## Research methods and design

For this literature study, we took an iterative approach for finding relevant literature. The Bachelor of Clinical Medical Practice (BCMP) programme at the University of Pretoria provided a list of published and unpublished articles regarding ClinAs. To identify further articles we systematically scanned the reference lists of all these articles. If a study looked promising we read the abstract and decided whether or not to include the article in our database. Each article that we included was searched in the same manner. This ‘snowballing’ approach was used until no further useful articles were identified. Articles were considered for inclusion when they were:

- Specifically about mid-level workers or ClinAs in South Africa, regardless of the specific subject
- Policy documents of the national government or provinces of South Africa regarding human resources for health or mid-level workers
- International reviews or meta-analyses regarding mid-level workers in developing countries
- International guidelines regarding mid-level workers in developing countries
- Directly applicable to one of our sub questions

In order to analyse costs of education and employment of ClinAs and MPs the University of Pretoria provided the tuition fees of the BCMP (clinical associate), MCBhB (medical practitioner) and BCUR (nurse) degree programmes. Besides that, an overview was obtained of the salary notches of healthcare workers in South Africa in 2014. The Department of Health and the Department of Education of the province Mpumalanga provided an overview of their budget for bursaries for students at the Faculty of Health Sciences at the University of Pretoria.

Each article was scrutinised to see if they answered one or more of our sub questions. The costs of the education and employing ClinAs and MPs were analysed in Microsoft Excel 2010.

The results were used to present a case study in the discussion section of this article.

## Ethical considerations

Ethical clearance for the study was obtained from the Research Ethics Committee of University of Pretoria (approval number: 56/2011).

## Results

### Sub question 1: What are the costs for a province to train a clinical associate compared with training a medical practitioner?

The provinces and NDoH funds grants and bursaries for students, so that they are able to follow a healthcare education. The bursaries are paid to the universities and cover the costs for fees, accommodation, meals and books. The students, who receive a bursary, must in return ‘work back’ their bursary obligation in the public sector after qualifying.^1,3^

A comparison was made between the bursaries for BCMP students at the University of Pretoria and the bursaries for MBChB students (Mpumalanga Department of Education 2015, personal communication, Oct 8; University of Pretoria 2015, personal communication, Oct 8) (Tables 1 and 2). The first is a three-year programme, while the latter is a six-year programme.

**TABLE 1:** Cost of BCMP (clinical associate) bursaries (in rand) for 2015.

Year	Fees†	Accommodation‡	Meals	Books	Total
1	50 285	34 200	28 800	6500	119 785
2	15 185	34 200	28 800	6500	84 685
3	26 880	34 200	28 800	6500	96 380
**Total**				**300 850**

*Source*: Mpumalanga Department of Education 2015

† The fees include tuition, study material, facility and copyright & library.

‡ Accommodation costs are in some individual cases higher, however never lower.

**TABLE 2:** Cost of MBChB (medical practitioner) bursaries (in rand) for 2015.

Year	Fees†	Accommodation‡	Meals	Books	Total
1	52 805	34 200	28 800	6500	122 305
2	54 455	34 200	28 800	6500	123 955
3	49 225	34 200	28 800	6500	118 725
4	50 115	34 200	28 800	6500	119 615
5	52 610	34 200	28 800	6500	122 110
6	54 775	34 200	28 800	6500	124 275
**Total**				**730 985**

*Source*: University of Pretoria 2015?,

† The fees include tuition, study material, facility and copyright & library.

‡ Accommodation costs are in some cases higher, however never lower.

The total cost for a province to pay for the full training of a clinical associate is R 300 850 and a medical practitioner R 730 985. This indicates that it will cost a province approximately two and a half times more to train a medical practitioner than a clinical associate at the University of Pretoria. The training of MPs in Cuba is more expensive and exceeds R 1 million per person.

### Sub question 2: What are the costs for a province to employ a clinical associate in comparison with employing a medical practitioner?

There are already calculations about the average cost of a medical practitioner,^2^ however, not yet of the average cost of a clinical associate as this is a new cadre. We therefore came up with a new calculation as an indication to compare the average cost to employ a clinical associate with that of a medical practitioner.

The main cost of an employee is his or her salary. Therefore, we compared the average salary of a clinical associate, for a 10-year period, with the average salary of a medical practitioner, for the first 10-year period of employment (Tables 3 and 4).^10^

**TABLE 3:** Average salary of Clinical Associate (in rand) in South Africa.

Year	Salary level	Full-time salary 01-Apr-14
1	PSAP (Non-OSD)† Level 7: no 1	183 438
2	PSAP (Non-OSD) Level 7: no 2	186 192
3	PSAP (Non-OSD) Level 7: no 3	188 985
4	PSAP (Non-OSD) Level 7: no 4	191 820
5	PSAP (Non-OSD) Level 7: no 5	194 694
6	PSAP (Non-OSD) Level 7: no 6	197 616
7	PSAP (Non-OSD) Level 7: no 7	200 577
8	PSAP (Non-OSD) Level 7: no 8	203 589
9	PSAP (Non-OSD) Level 7: no 9	206 640
10	PSAP (Non-OSD) Level 7: no 10	209 739
**Average**		**196 329**

† PSAP (Non-OSD) = public service act appointees not covered by occupation-specific dispensation (OSD).

**TABLE 4:** Average salary of Medical practitioners (in rand) in South Africa.

Year	OSD post	Full-time salary 01-Apr-14
1	Medical Officer (Intern)	357 237
2	Medical Officer (Intern)	362 595
3	Medical Officer (Community Service)	478 977
4	Medical Officer (Gr 1)	596 118
5	Medical Officer (Gr 1)	605 067
6	Medical Officer (Gr 1)	614 136
7	Medical Officer (Gr 1)	623 346
8	Medical Officer (Gr 1)	632 697
9	Medical Officer (Gr 1)	642 192
10	Medical Officer (Gr 2)	681 603
**Average**		**559 397**

**FIGURE 1:**
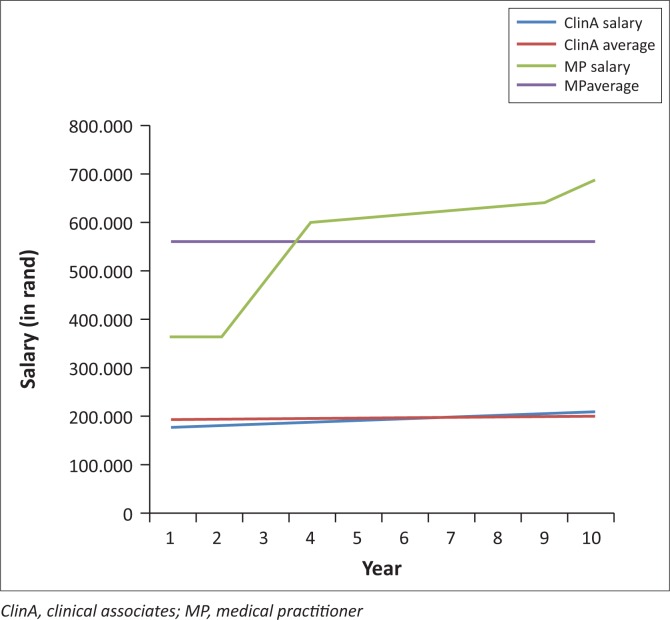
Salary cost clinical associates and medical practitioners.

Based on the above calculation, the total cost for a province to pay for employment of one clinical associate in the public service is on average R 196 329 per year and to employ a medical practitioner in the public service is on average R 559 397 per year for the first 10 years after graduation. This excludes any overtime remuneration and indicates that it will cost nearly three times more for a province to employ a medical practitioner in public service than a clinical associate. Because of the fast rise in salaries for MPs in the first two years (Figure 1), this difference is expected to become larger when looking at more senior MPs compared with more senior ClinAs.

### Sub question 3: What is the scope of practice of clinical associates and how does this compare to medical practitioners?

When looking at the scope of practice for the medical profession, it is clear that MPs in South Africa are allowed to practise medicine in the broadest sense of the word.^11^

The scope of practice of ClinAs on the other hand is much more specifically defined in the *Health Profession Act*.^12^ In summary, ClinAs are only allowed to practice under continuous supervision in accordance to their level of education, training and experience. Furthermore, they are allowed to do a number of specifically defined procedures described in the *Health Profession Act*.^12^

They can also perform *any act* delegated by the supervising medical practitioner in accordance with the education, training and experience of the clinical associate. They cannot conduct an independent private practice; they cannot act as a substitute for a medical practitioner.^12^

### Sub question 4: How much medical practitioner time can a clinical associate save?

In Rwanda, there has been a study to quantify medical practitioner time saved through task shifting.^13^ The demand on medical practitioner time was reduced by 76% when nurses were used to take over tasks of MPs.^13^

In this pilot study, nurses were trained to function specifically in an HIV programme. It seems a safe assumption that training ClinAs for similar routine tasks would yield similar results. However, one could argue that ClinAs in district hospitals perform a wider variety of tasks than in the above-mentioned study and will therefore achieve a smaller reduction of medical practitioner time.

Therefore, we will work with two different scenarios in the case study. In the first scenario, we will use a medical practitioner reduction time of 76%.

In the second scenario, we will use a physician reduction time of 50% to compensate for the lack of knowledge in this area. It seems reasonable that ClinAs are able to at least free up 50% of the medical practitioners’ time.^4,5,6,8^ In a recent study by Ngcobo on voluntary male circumcision by clinical associates and doctors he found that there was no difference in quality and productivity between the two groups.^14^ An American study of physician assistants found that 70–90% of the work of a primary care physician can be done by a non-physician clinician (NP-C).^15^

### Sub question 5: What is the quality of care provided by clinical associate?

There has been one very recent study in South Africa that found that ClinAs provide the same quality of care and outcome compared to MP`s when doing Voluntary Male Medical Circumcisions at a much lower cost per circumcision.^14^

A number of literature reviews, international guidelines and even a meta-analysis have also looked at the quality of care provided by mid-level health workers (MLWs).^4,5,6,7,8^

The general consensus seems to be that although more evidence is needed in African countries, mid-level workers can provide the same quality of care as higher level workers provided that they receive adequate training, support and supervision. If this is not provided, the quality of care can be sub optimal.^4,5,6,7,8^

### Sub question 6: How is the retention of clinical associates in rural areas and how does this compare to medical practitioners?

ClinAs are more willing to work in rural areas compared to MPs.^1^ A study that tracks the first 250 BCMP graduates from the University of Pretoria found that 57% work in the rural public health institutions (personal communication Lekgala Monareng Master’s thesis, 2016). Reasons are that they are recruited from rural communities and are trained close to where they are recruited. Furthermore, their degree is not recognised internationally.^3,6,7,9^

One study done at the University of Pretoria found that if given complete freedom of choice without bursary or family obligations 53.4% of the BCMP students indicated a preference to work in rural areas.^1^ For medical students this percentage was 4.8%.^1^

However, one must not assume that mid-level workers will tolerate low salaries and poor living and working conditions. Low pay, poor supervision and lack of training are associated with lower retention.^5,7^ Therefore, good salary and incentive structures must be developed and implemented.^5,6,8^

## Discussion

The discussion will consist of a case study for the province of Mpumalanga, general considerations regarding the training and employing of ClinAs and the limitations of this study.

### Case study: Mpumalanga

In 2010, the province Mpumalanga had 535 vacant posts in the public sector for MPs.^2^ Suppose that the province decides to invest an extra budget of R 10 000 000 in training healthcare workers in order to fill these vacancies for MPs. We will investigate the cost effectiveness of two different strategies to spend this extra training budget:

**Scenario 1**: In this scenario, the extra budget is used to train only MP’s.

**Scenario 2a**: The extra budget is used to train a combination of ClinAs and MP’s; ClinAs can free up 50% of the MPs time.

**Scenario 2b**: The extra budget is used to train a combination of ClinAs and medical practitioners; ClinAs can free up 76% of the MP’s time.

In scenarios 2a and 2b we will use a training and employing ratio of 1:2. For every medical practitioner trained and employed, two ClinAs are trained and employed.

It is important to realise that ClinAs free up time for MPs, so that these MPs can do the work of two or more vacant MP posts. Also, this case study looks at the best strategy if a province frees up *extra* budget to train. The training and employing of ClinAs should not replace or compete with the current training and employment of MP’s.

### Scenarios

Based on the costs for bursaries and the average salaries we calculated the costs for the province to train and employ the MPs and ClinAs for the different scenarios. Also the cost of every filled vacant MP post per year was calculated. The results are summarised in Table 5. Note the difference in costs per filled vacant MP post between scenario 2a and 2b because more vacant MP posts can be filled in scenario 2b.

**TABLE 5:** Summary case study.

Variable	Assumption	Extra budget allows to train†	This will fill potentially	Total employment costs‡	Costs per filled vacant MP post
Train MPs	None	13.7 clinicians:	13.7 vacant MP posts	R 7.7 000 000 per year	R 559 397 per year
		13.7 MPs			
		0 CAs			
Train MPs and ClinAs	’*CA can free up 50% MP time*’	22.5 clinicians:	15 vacant MP posts	R 7.1 000 000 per year	R 476 027 per year
		7.5 MPs			
		15 CAs			
Train MPs and ClinAs	’*CA can free up 76% MP time*’	22.5 clinicians:	18.9 vacant MP posts	R 7.1 000 000 per year	R 377 800 per year
		7.5 MPs			
		15 CAs			

Province Mpumalanga: 585 vacant posts for MPs in public sector in 2010. Suppose: the province decides to invest 10 million rand extra in training more healthcare workers.ClinAs, Clinical Associates; MPs, medical practitioners.

† Based on bursaries for BCMP and MBChB students; see Table 1.

‡ Based on average salary for Clinical Associates and MPs; see Table 2.

To summarise the case: training and employing ClinAs are effective because they can free up time of MPs, which the MPs can spend to fill more vacant MP posts, without loss of quality of care. It is also cheaper to train and employ a combination of MPs and ClinAs, than only training and employing MPs. How much cheaper depends on the productivity of the ClinAs.

We therefore conclude that it is a more cost-effective strategy for the province of Mpumalanga to spend the extra budget on training a combination of MPs and ClinAs than on spending it only on training MPs.

## Considerations

To our knowledge this report is the first that investigates the cost effectiveness of ClinAs in South Africa. The literature review that has been done was extensive. Our data regarding the costs and employment are ‘hard data’ straight from the source. We believe that this report provides a good base for further and more extensive studies regarding the cost effectiveness of ClinAs in South Africa.

Training and employing ClinAs can potentially be a cost-effective strategy for a province to meet the demand for health workers in rural areas. However, in order to ensure that this strategy is successful some considerations must be taken into account.

Besides allocating money for the extra training of ClinAs, there should also be funding to employ them afterwards. According to the NDoH this is not always the case.^2^

Another consideration is that the use of ClinAs is not a ‘magic bullet’ solution. It is one strategy in a range of strategies to tackle the shortage of healthcare workers.^6,7,8^ Neither should they be seen as an ‘investment free’ solution who will tolerate poor working and living conditions and low salaries.^7^

## Limitations

A limitation of this study is the lack of research done in the area of mid-level workers in developing countries. Therefore, assumptions had to be made regarding the MP time saved in a district hospital.

Also little is known about the actual retention and if bursary obligations are honoured after graduation.

We used the average salary to give a general indication for the cost of employment; however, this might not reflect the complete costs for the province.

## Conclusion

The training and employment of ClinAs are a cost-effective strategy for a province to meet the increasing demand for healthcare workers in rural areas. ClinAs can perform routine tasks for MPs, without losing quality of care, and therefore free up time for MPs. Costs, per filled vacant MP post, of training and employing a combination of ClinAs and MPs are less than only training and employing MPs. This strategy will, however, only succeed if ClinAs receive adequate training, support and supervision and if the province keeps investing in them.
